# In Utero Alcohol Exposure, Epigenetic Changes, and Their Consequences

**DOI:** 10.35946/arcr.v35.1.05

**Published:** 2013

**Authors:** Michelle Ungerer, Jaysen Knezovich, Michele Ramsay

**Affiliations:** **Michelle Ungerer***is a Masters student and lecturer in the Division of Human Genetics, School of Pathology, Faculty of Health Sciences, University of the Witwatersrand and the National Health Laboratory Service in Johannesburg, South Africa.*; **Jaysen Knezovich, M.Sc.,***is a Ph.D. student and lecturer in the Division of Human Genetics, School of Pathology, Faculty of Health Sciences, University of the Witwatersrand and the National Health Laboratory Service in Johannesburg, South Africa.*; **Michele Ramsay, Ph.D.,***is a professor in the Division of Human Genetics, National Health Laboratory Service, School of Pathology, Faculty of Health Sciences, and Interim Director of the Syndey Brenner Institute for Molecular Bioscience at the University of the Witwatersrand, Johannesburg, South Africa.*

**Keywords:** Prenatal alcohol exposure, fetal alcohol spectrum disorders, epigenetics, epigenetic mechanisms, epigenetic modifications, brain development, cognitive deficits, behavioral deficits, DNA methylation, histone modification, chromatin, microRNAs, rodent models

## Abstract

Exposure to alcohol has serious consequences for the developing fetus, leading to a range of conditions collectively known as fetal alcohol spectrum disorders (FASD). Most importantly, alcohol exposure affects the development of the brain during critical periods of differentiation and growth, leading to cognitive and behavioral deficits. The molecular mechanisms and processes underlying the teratogenic effects of alcohol exposure remain poorly understood and are complex, because the specific effects depend on the timing, amount, and duration of exposure as well as genetic susceptibility. Accumulating evidence from studies on DNA methylation and histone modification that affect chromatin structure, as well as on the role of microRNAs in regulating mRNA levels supports the contribution of epigenetic mechanisms to the development of FASD. These epigenetic effects are difficult to study, however, because they often are cell-type specific and transient in nature. Rodent models play an important role in FASD research. Although recent studies using these models have yielded some insight into epigenetic mechanisms affecting brain development, they have generated more questions than they have provided definitive answers. Researchers are just beginning to explore the intertwined roles of different epigenetic mechanisms in neurogenesis and how this process is affected by exposure to alcohol, causing FASD.

Alcohol exposure of the developing embryo and fetus in utero can have a wide range of detrimental effects collectively referred to as fetal alcohol spectrum disorders (FASD). Researchers are intensively investigating the mechanisms that may contribute to alcohol’s effects on the developing organism and to the resulting consequences, particularly with respect to the cognitive and behavioral deficits associated with FASD. These studies have yielded increasing evidence that epigenetic mechanisms play an important role in these processes. This article reviews the current knowledge regarding the contributions of epigenetic modifications to the manifestations of FASD, much of which has been obtained using rodent models in which the timing, frequency, duration, and amount of alcohol exposure can be tightly controlled. This discussion also touches on the concepts of developmental reprogramming, the role of preconception alcohol exposure, and transgenerational transmission of the effects of alcohol exposure.

## FASD

FASD can be associated with a variety of symptoms that differ widely in severity depending on the specific conditions of alcohol exposure. The most severe outcome is fetal alcohol syndrome (FAS), which can manifest variably with diverse combinations of craniofacial, growth, central nervous system (CNS), and neurobehavioral abnormalities (Jones et al. 1973; Sampson et al. 1997). Associated psychosocial problems include learning difficulties, attention deficit–hyperactivity disorder (ADHD), and mental retardation (Burd et al. 2003; O’Leary 2004). Given that alcohol consumption is voluntary, FASD is said to be the most preventable cause of birth defects and mental retardation. FASD is a global health concern, and worldwide approximately 1 to 3 per 1,000 births is thought to be suffering from FAS. In the United States, FAS prevalence ranges between 0.5 and 2.0 per 1,000 live births (Abel 1995; May and Gossage 2001). The highest rates of FAS have been reported in mixed-ancestry communities in the Western Cape of South Africa, where between 68.0 and 89.2 per 1,000 school-age children display FAS symptoms (May et al. 2007).

Between 5 and 10 percent of offspring who have been exposed to alcohol prenatally display alcohol-related developmental anomalies (Abel 1995), with the severity of the outcome determined by the dose, timing, and duration of exposure (Padmanabhan and Hameed 1988; Pierce and West 1986; Sulik 1984). However, the proportion of affected offspring may be considerably higher in unfavorable circumstances, including instances of malnutrition of the mother and thus, the fetus. The genetic makeup of both mother and fetus, in conjunction with other factors (e.g., gender, diet, and social environment), also plays an important role in the manifestation of FASD (Chernoff 1980; Ogawa et al. 2005).

The effects of prenatal alcohol exposure are more similar in identical (i.e., monozygotic) twins than in fraternal (i.e., dizygotic) twins, suggesting a heritable component (Abel 1988; Chasnoff 1985; Streissguth and Dehaene 1993). Genetic studies have shown that different variants of the genes encoding various alcohol-metabolizing enzymes— such as alcohol dehydrogenases (ADHs), aldehyde dehydrogenases (ALDHs), and cytochrome P450 2E1 (CYP2E1)—in the mother and their offspring can affect alcohol metabolism and contribute to subsequent alcohol-related damage (Gemma et al. 2007; Warren and Li 2005). For example, variants at the ADH1B locus that result in an altered amino acid sequence and function of the encoded enzyme can influence the severity of the adverse effects on the developing fetus (i.e., teratogenesis) in different ethnic populations (for a review see Ramsay 2010). However, to date few studies have supported a role for genetics in the development of FASD.

Rodent models have provided a valuable tool for investigating genetic influences on the observable outcomes (i.e., phenotypes) associated with FASD. For example, the effects of in utero alcohol exposure differ between inbred and selectively bred mice. These findings highlight the contribution of a genetic predisposition to the susceptibility to the detrimental effects of prenatal ethanol exposure and provide additional support for the importance of genetic factors in the development of FASD (Boehm et al. 1997; Gilliam et al. 1989; Ogawa et al. 2005).

Although studies have investigated the genetic susceptibility to FASD, the underlying cause(s) of these disorders still remains unclear. The wide range of clinical features observed in people affected by in utero alcohol exposure underlines the importance of investigating the mechanisms of alcohol-related teratogenesis at a molecular level. Because FASD is a developmental abnormality, disruptions in normal cellular differentiation driven by changes in gene expression that in turn are regulated by epigenetic mechanisms are most likely involved in FASD pathogenesis.

## Epigenetic Modifications

The term epigenetics, first defined by Waddington in 1942 (as reprinted in Waddington 2012), refers to the changes in gene expression that occur without changes in the DNA sequence itself. Epigenetics plays a vital role in regulating key developmental events, allowing for tissue-specific gene expression, genomic imprinting,^1^ and stem-cell maintenance. Tissue-specific gene expression patterns are established and maintained through two mechanisms; structural chromatin modifications (i.e., DNA methylation and histone modifications) and RNA interactions (i.e., the actions of non-coding RNAs [ncRNAs]). In eukaryotes, the genome is present in the cell nucleus in the form of chromatin—a DNA–protein complex that packages DNA into a highly condensed form. The structural building blocks of chromatin are the nucleosomes, each of which consists of 147 base pairs of DNA wrapped around a core of 8 histone proteins (Ooi and Henikoff 2007). The octamer core comprises two copies each of histone proteins H2A, H2B, H3, and H4. Moreover, the nucleosomes are connected with each other by a linker histone H1 that offers stability to the packaged structure. Modifications of the chromatin structure affect the first step of gene expression (i.e., transcription). ncRNAs, on the other hand, act at the posttranscriptional level.

### Chromatin Remodeling

#### DNA Modifications

Both DNA and protein components of the nucleosome are subject to a variety of modifications that can influence chromatin conformation and accessibility. The best-characterized epigenetic mark, DNA methylation, involves the covalent addition of a methyl (CH_3_) group to one of the four DNA nucleotides (i.e., cytosine [C]) to form 5-methylcytosine (5mC). In eukaryotes, methylation usually affects C that are followed by the nucleotide guanine (G) (i.e., that are part of a CpG dinucleotide) (Rodenhiser and Mann, 2006). At these sites, enzymes called DNA methyltransferases (DNMTs) mediate the methylation of C residues, thereby acting as critical modulators of fetal development (Li et al. 1992). For these DNA methylation reactions, DNMTs use methyl groups produced by a sequence of reactions known as the folate pathway (Friso et al. 2002). Generally, DNA methylation is associated with a condensed chromatin conformation, which effectively silences gene expression because the enzymes needed for transcription cannot access the DNA.

More recent studies have found that 5mC can be further modified by enzymes called ten-eleven translocation (Tet) proteins, in a process referred to as iterative oxidation. This results in the formation of several reaction products (i.e., derivatives), including 5-hydroxymethylcytosine (5hmC), 5-formylcytosine (5fC), and 5-carboxylcytosine (5caC) (Ito et al. 2011; Tahiliani et al. 2009). Although the role of these methylation derivatives still remains unclear (Branco et al. 2012) they seem to serve different functions than 5mC. Thus, the conversion of 5mC to 5hmC has been implicated in active DNA demethylation (Wu and Zhang, 2010). Furthermore, whereas 5mC typically is found in regions regulating the expression of specific genes (i.e., in promoters), 5hmC is associated with the bodies of the affected genes or with promoters of developmental regulatory genes (Wu et al. 2011). Finally, 5hmC appears to play an important role in reprogramming the paternal genome following fertilisation (Hackett and Surani 2013). (Reprogramming will be discussed in the following section.)

#### Histone Modifications

The histones making up the core of the nucleosome have unstructured N-terminal tails that protrude from the nucleosome and which are subject to modifications. Histone modifications are varied and include acetylation, methylation, phosphorylation, ubiquitinylation, ADP-ribosylation, and sumoylation at specified residues (for a review, see Kouzarides 2007). Importantly, these modifications are dynamic—that is, they can be removed again by specific enzymes.

These histone modifications, together with DNA methylation, influence chromatin structure and have a profound influence on gene regulation. Both of these types of epigenetic modifications work together to remodel the chromatin and partition the genome into two different functional domains—transcriptionally active regions collectively known as euchromatin and transcriptionally inactive regions collectively called heterochromatin. Euchromatic regions are modified to allow an open conformation, rendering the regions accessible to cellular proteins favoring transcription. In contrast, heterochromatic regions, such as the ends of chromosomes (i.e., telomeres) and regions around the center of the chromosome (i.e., pericentric regions), generally exhibit a closed conformation that limits interactions between the DNA and cellular proteins, thereby silencing gene activity (for a review, see Schneider and Grosschedl 2007). Additionally, chromatin structure, and thus gene expression, is influenced by the specific combination of histone variants in a nucleosome, the spacing between nucleosomes (i.e., nucleosome occupancy), and the position of each nucleosome within the nucleus (i.e., nuclear architecture) (Cairns 2009).

## Developmental Reprogramming

**Figure 1:**
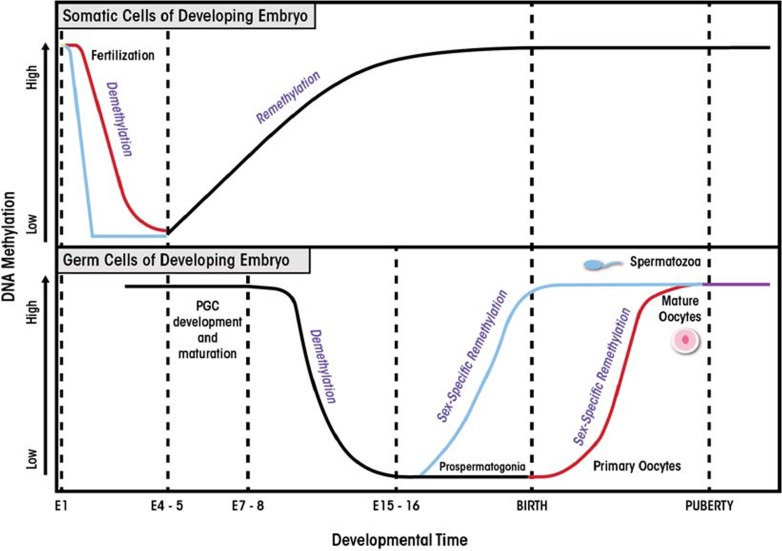
Reprogramming in mammalian development. Two waves of epigenetic reprogramming occur during embryo development. The first phase of reprogramming occurs in the normal body cells (i.e., somatic cells) of the developing embryo. In mice, following fertilization, the embryo undergoes genome-wide demethylation that is completed by embryonic day 5 (E5). The paternal genome (blue line) undergoes rapid, active demethylation, whereas in the maternal genome (pink line), demethylation occurs via a passive process. Remethylation of the embryonic genome begins at day E5 and is completed prior to birth. The second wave of epigenetic reprogramming occurs in the germ cells of the developing embryo, which will ultimately give rise to gametes that contain sex-specific epigenetic signatures. The primordial germ cells (PGCs) of the developing embryo contain the methylation signatures of the parental genomes. At approximately E7–8, the PGCs undergo rapid demethylation that is complete by E15–16. Following this, sex-specific methylation is re-established. In the male germline, reprogramming is complete at birth (blue line), whereas in females, reprogramming continues until puberty (pink line). SOURCE: Adapted from Reik et al. 2001; Smallwood and Kelsey 2012.

Epigenetic reprogramming is a process that involves the erasure and then re-establishment of chromatin modifications during mammalian development. It serves to erase random changes in epigenetic marks (i.e., epimutations) that have occurred in the germ cells (i.e., gametes) and to restore the ability of the fertilized egg cell (i.e., zygote) to develop into all the different cell types and tissues (Reik et al. 2001). Epigenetic modifications are modulated in a temporal and spatial manner and act as reversible switches of gene expression that can lock genes into active or repressed states. In addition, these modifications allow the zygote to give rise to the cellular lineages that will form the embryo. Reprogramming occurs in two phases during in utero development, one shortly after fertilization and the other in the developing gametes of the fetus. The first phase takes place after fertilization in the preimplantation embryo (i.e., the blastocyst). During this phase, embryonic epigenetic patterns are re-established in a lineage-specific manner in the inner cell mass of the blastocyst (figure 1). The second phase occurs in the gametes, where rapid genome-wide demethylation is initiated to erase existing parental methylation patterns, followed by re-establishment of epigenetic marks in a sex-specific manner (Reik et al. 2001).

Researchers recently have begun to investigate epigenetic mechanisms as key contributors to the development of FASD. This research was prompted by the observation that periods of increased vulnerability to in utero alcohol exposure coincide with those of reprogramming events. In addition, evidence suggests that environmental factors, and specifically alcohol, are able to alter epigenetic modifications. This provides a link between the genotype, environment, and disease.

## Alcohol and Biological Pathways

As mentioned previously, DNA methylation reactions rely on the folate pathway to supply the necessary methyl groups. Excessive alcohol exposure is known to interfere with normal folate metabolism and reduce its bioavailability (Halsted and Medici 2012). Folate is required as a coenzyme to supply methyl groups needed for the formation of a compound called S-adenosylmethionine (SAMe), which in turn participates in reactions in which the methyl group is transferred to another molecule (i.e., transmethylation reactions). In the folate-dependent pathway, the enzyme methionine synthase (MS), which requires vitamin B12 to function properly, is responsible for transferring the methyl group contained within the 5-methyl-tetrahyrofolate compound to homocysteine, which ultimately generates methionine (Friso et al. 2002). The methionine is converted to SAMe by methionine adenosyltransferase (MAT), and the SAMe then is used for the methylation of DNA. As early as 1974, research on alcohol-fed rats described reduced MS activity and subsequent reduction of the levels of both methionine and SAMe (Barak et al. 1987; Finkelstein 1974; Trimble et al. 1993). Additionally, ethanol appears to enhance the loss of methyl groups, which in turn disrupts subsequent SAMe-dependent transmethylation reactions (Schalinske and Nieman 2005).

## Rodent Models of Prenatal Ethanol Exposure

The teratogenic effects of prenatal alcohol exposure have been examined in rodent models for several decades. Studies have shown that in utero exposure to alcohol in these animals results in a wide range of anomalies, including growth retardation, CNS malformations, mental disability, and distinct craniofacial dysmorphology (Anthony et al. 2010; Boehm et al. 1997; Boggan et al. 1979; Bond and Di Giusto 1977; Klein de Licona et al. 2009; McGivern 1989; Parnell et al. 2009).

The FASD-like phenotypes observed in these rodent models have been associated with alterations in global gene expression, particularly in the developing brain (Hard et al. 2005; Hashimoto-Torii et al. 2011, 2011; Kleiber et al. 2012). This association, in conjunction with the vital role that epigenetic mechanisms play in controlling gene expression, suggests that normal epigenetic regulation by DNA methylation, histone modifications, and ncRNAs is disrupted as a result of ethanol insult.

### Prenatal Ethanol Exposure and DNA Methylation

A direct link exists between ethanol exposure and aberrations in DNA methylation. For example, in a mouse model evaluating the effects of in utero ethanol exposure from days 9 to 11 of gestation, this acute ethanol administration resulted in lower-than-normal methylation throughout the genome (i.e., in global hypomethylation) of fetal DNA (Garro 1991). Furthermore, the ethanol-exposed fetuses displayed significantly reduced levels of DNA methylase activity. Ethanol-induced reductions in DNA methylation affect not only the fetus but also the placenta in pregnant mice exposed to alcohol (Haycock and Ramsay 2009). More recently, researchers evaluated the effect of prenatal alcohol exposure on DNA methylation of five imprinted genes in male offspring; these analyses detected a decrease in DNA methylation at a single locus in the *H19* imprinting control region in the sperm of these males (Stouder et al. 2011). Finally, in utero ethanol exposure in mice hinders the acquisition of DNA methylation in a brain region called the dentate gyrus, which is associated with developmental retardation (Chen et al. 2013).

Other analyses have looked at methylation patterns of specific genes rather than global DNA methylation. For example, a gene called *Agouti* has been used extensively as a model to study the effects of environmental (i.e., dietary) exposures on DNA methylation. The murine *Agouti* (*A*) locus regulates the animals’ coat color; animals that carry two copies of the common variant, referred to as the wild-type allele, (i.e., *a/a* mice) display a pseudoagouti (i.e., brown) coat. A gene variant called *A^vy^* is a dominant mutation that is caused by the insertion of a DNA sequence known as an intracisternal A-particle (IAP) retrotransposon in front of (i.e., upstream of) the *Agouti* gene. Animals that carry one mutant and one wild-type gene copy (i.e., heterozygous *A^vy^/a mice*) display a variety of coat colors, ranging from yellow to mottled and brown, even though they are genetically identical. *A^vy^* expression is strongly correlated to the DNA methylation profile of the inserted IAP. If the IAP shows hypomethylation, the *Agouti* gene is constantly expressed (i.e., shows constitutive ectopic *Agouti* expression) and the animals have a yellow coat. Conversely, hypermethylation correlates with promoter silencing and a pseudoagouti coat (Dolinoy et al. 2010). Kaminen-Ahola and colleagues (2010) investigated the effect of gestational ethanol exposure in *A^vy^* heterozygous mice, demonstrating that ethanol exposure increased the proportion of pseudoagouti-colored offspring. This change in the proportion of coat colors was linked to transcriptional silencing of the mutant allele, which in turn correlated with hypermethylation of the *A^vy^* locus. This study highlights the ability of prenatal alcohol exposure to alter the fetal epigenotype (albeit only at a specific locus) and, consequently, the adult phenotype.

In addition to the aberrant expression at the *Agouti* locus in the *A^vy^* heterozygous mice, Kaminen-Ahola and colleagues (2010) noted altered gene expression profiles in the livers of their ethanol-exposed wild-type (*a/a*) siblings, as well as growth restriction and certain craniofacial dysmorphologies that are reminiscent of human FAS symptoms. Together, the findings that ethanol exposure can alter DNA methylation at the *Agouti* locus and elicit an associated phenotype (i.e., altered coat color), suggests that other epigenetic targets and associated gene expression also may be disrupted and may be responsible for the occurrence of a phenotype that corresponds to FAS in humans.

Similar studies have demonstrated the association of ethanol exposure with changes in DNA methylation and concurrent alterations in the expression of other genes. Downing and colleagues (2010) found that in utero ethanol exposure resulted in reduced methylation in the embryo at the *Igf2* locus, which encodes insulin-like growth factor 2, with a concomitant change in *Igf2* gene expression. These changes in gene expression were accompanied by skeletal malformations similar to those observed in FAS patients. In other studies, alcohol exposure resulted in neural tube defects in conjunction with genome-wide bidirectional methylation changes (i.e., occurrence of both hypo- and hypermethylation) (Liu et al. 2009). These altered methylation profiles were associated with significant changes in the expression of several genes associated with multiple functions, including chromatin remodeling, neuronal morphogenesis, synaptic plasticity, and neuronal development.

Together, these findings provide compelling evidence for alcohol-induced alterations of DNA methylation patterns in exposed fetuses that elicit a phenotype that is at least in part similar to that observed in FASD.

### Prenatal Ethanol Exposure and Histone Modifications

Rodent models of alcoholism and in utero exposure to ethanol, as well as studies using cultured cells (i.e., in vitro experiments) have provided significant insights into the effects of alcohol on protein modifications, particularly to histones. Excess alcohol intake can exert its effect on protein modifications either directly or indirectly by disrupting the epigenetic machinery.

As with DNA methylation, some of these mechanisms involve folate, which as mentioned earlier serves as methyl group donor for histone methylation. Folate deficiency is a common clinical sign of chronic alcohol abuse and has been implicated in the development of alcoholism-related complications, such as alcoholic liver disease (Eichner et al. 1971). These deficiencies have been associated with significant alterations in histone modifications, particularly at lysine residues (Esteller 2008; Kim and Shukla 2005; Park et al. 2003; Shukla et al. 2008). Altered histone modification, in turn, is associated with altered gene expression (Pal-Bhadra et al. 2007).

In in vitro studies using cultured rat liver cells (i.e., hepatocytes), ethanol exposure has been associated with bidirectional changes in histone methylation, including increased methylation at lysine 4 of histone H3 (i.e., increased H3K4me2) and decreased methylation at lysine 9 of histone H3 (i.e., decreased H3K9me2) (Pal-Bhadra et al. 2007). In addition, ethanol exposure led to selective acetylation of H3K9 (Park et al. 2003). These findings have been supported by in vivo models that have demonstrated increased H3K9 acetylation in the liver, lung, and spleen of adult rats acutely exposed to alcohol (Kim and Shukla 2006). Chronic alcohol exposure in adult rats also has been associated with increases in histone H3 and H4 acetylation in the amygdala of the brain that subsequently led to changes in the expression of the gene encoding a signaling molecule known as neuropeptide Y (Pandey et al. 2008). This increase in acetylation may result either from a decrease in the activity of the enzyme that removes acetyl groups (i.e., histone deacetylase) or an increase in the activity of the enzyme that adds acetyl groups (i.e., histone acetylase). Finally, in utero models have revealed that embryos exposed to acute levels of alcohol at mid-gestation showed elevated H3K9/18 acetylation as well as increased programmed cell death, referred to as apoptosis, of the fetal lung (Wang et al. 2010).

Zhong and colleagues (2010) investigated the effects of high and low levels of alcohol exposure on H3 acetylation and subsequent expression of genes related to heart development (i.e., *GATA4, Mef2c*, and *Tbx5*) in cardiac progenitor cells. Results indicated that low levels of alcohol increased H3 acetylation but did not significantly change the expression of the heart-development–related genes. In contrast, high levels of alcohol induced both H3 acetylation and significant gene-expression changes. These findings suggest that alterations to histone modifications are a potential mechanism for alcohol-induced cardiac disease (Zhong et al. 2010). An additional study by Guo and colleagues (2011) assessed the effects of alcohol on histone modifications in the cerebellum. The investigators found that perinatal alcohol exposure decreased the expression and function of one type of histone acetyl transferase called CREB binding protein (CBP). Altered CBP function resulted in decreased lysine acetylation on histones H3 and H4 within the cerebellum, which may contribute to the motor-activity deficits observed in FAS/FASD patients.

More recently, researchers investigated the effects of alcohol exposure on certain fetal neuronal stem cells (i.e., fetal cerebral cortical neuroepithelial stem cells) and associated gene expression. These analyses found that ethanol exposure led to significant reductions in the levels of H3K4me3 (which activates gene expression) and H3K27me3 (which represses gene expression) (Veazey et al. 2013). Despite the reduction in expression-activating H3K4me3 levels, both increased and decreased transcription was observed in the genes investigated. Furthermore, loss of the repressive methylation mark, H3K27me3, did not result in altered transcription levels.

Altered protein modifications in response to alcohol exposure also may involve proteins other than histones that contribute to other manifestations of FAS, including proteins involved in insulin signaling. People with FAS often exhibit an underdeveloped cerebellum (i.e., cerebellar hypoplasia) that is associated with impaired insulin-stimulated survival signaling. This impaired signaling is mediated by the body’s inability to properly respond to insulin (i.e., insulin resistance) (Soscia et al. 2006). It has been posited that chronic in utero ethanol exposure produces both insulin resistance in the CNS and oxidative stress, which is thought to play a major role in alcohol-related neurobehavioral terato-genesis (de la Monte and Wands 2010). In an in vivo model, adult rats prenatally exposed to alcohol exhibited reduced insulin signaling and increased expression of genes that regulate insulin (i.e., genes encoding proteins called TRB3 and PTEN) in the liver (Yao and Nyomba 2008). The analyses further suggested that the observed hepatic insulin resistance induced by alcohol exposure was associated with reduced acetylation of the TRB3 and PTEN proteins.

Taken together these findings suggest that alcohol-induced protein, and particularly histone, alterations continue to provide alternative or additional layers of complexity to an epigenetic etiology for FASD.

### Prenatal Ethanol Exposure and ncRNA Dysregulation

Another epigenetic mechanism by which alcohol could exert an effect on the epigenome is though the action of microRNAs (miRNAs). These small ncRNAs play a critical role in several key biological processes, especially during in utero development, including cell-cycle regulation, differentiation, and organ formation (i.e., organogenesis). Individual miRNAs can affect many target genes, silencing their expression either by preventing translation of the intermediate molecules (i.e., messenger RNAs [mRNAs]) that are generated during transcription or by causing mRNA cleavage. Experimental evidence indicates that the expression of miRNAs is altered following exposure to alcohol during development, and this may be one of the mechanisms underlying alcohol-related teratogenesis (Sathyan et al. 2007; Wang et al. 2009).

miRNAs have been implicated in the development of brain damage in response to prenatal alcohol exposure. Miranda and colleagues (2010) have hypothesized that ethanol causes brain damage during development by promoting the cell cycle of neural stem cells. This would accelerate the maturation of these progenitor cells and result in their premature depletion. This hypothesis is compatible with the observation that when clusters of neural stem cells (i.e., neurospheres) are grown in culture, differentiating neuroblasts from these clusters show increased migration and depletion of stem cells when they are exposed to ethanol compared with their unexposed counterparts. This observed behavior suggests the involvement of a large network of genes controlling complex biological outcomes. In order to examine the trigger for this behavior, researchers examined miRNA expression levels in alcohol-exposed and nonex-posed neural stem cells. A preliminary screen of miRNAs in neural stem cells identified four miRNAs (i.e., miR9, miR21, miR135 and miR355) that were suppressed in the presence of ethanol exposure (Sathyan et al. 2007). These miRNAs were found to act both antagonistically and synergistically, both reducing and promoting apoptosis. Normally, these miRNAs favor normal development by balancing cell survival and cell proliferation. Following alcohol exposure, however, the reduction in their levels leads to an imbalance with detrimental effects.

In another study (Wang et al. 2009), pregnant mice were exposed to ethanol from day 6 to day 15 of gestation, and fetal brain tissue was examined for differential miRNA expression. Under these conditions, seven miRNAs were upregulated and eight were downregulated in response to ethanol exposure, with miR10a and miR10b showing the highest level of overexpression. It is biologically plausible that overexpression of these two miRNAs can disrupt developmental processes because they are thought to regulate expression of a group of genes called the *Hoxb* gene family (Wang et al. 2009). This group of genes is involved in the regulation and establishment of body patterning during embryonic development. Interestingly, there was no overlap in the miRNAs between this study and those identified in the study by Sathyan and colleagues (2007), suggesting that different models for alcohol exposure as well as the investigation of different tissues and different developmental time periods of exposure may have varying impacts on diverse miRNA targets.

Taken together, the preliminary studies suggest that miRNA plays a crucial role in normal development and that this process can be disrupted by alcohol exposure during critical periods, especially during neurogenesis.

## Role of Preconception Alcohol Exposure in FASD

Although studies of FASD etiology predominantly have focused on maternal exposure during pregnancy, evidence also exists in support of contributions of paternal exposure. For example, FAS-like effects have been observed in children of alcoholic fathers even in the absence of gestational alcohol exposure, suggesting the possibility that preconception alcohol exposure may affect offspring development (Abel and Tan 1988; Lemoine et al. 1968). Studies conducted in rodents 100 years ago have supported these findings (Stockard 1913; Stockard and Papanicolaou 1916), and more recent analyses also reported that paternal preconception alcohol exposure was associated with neurobehavioral abnormalities, low birth weights, congenital malformations, and growth retardation in offspring (Friedler 1996; Jamerson et al. 2004). Additional studies have implicated a role for altered sperm DNA methylation in paternally-mediated effects of preconception ethanol exposure on offspring development (Knezovich and Ramsay 2012).

## Transgenerational Transmission of the Effects of Alcohol Exposure

Altered epigenetic modifications (i.e., epimutations) may also be passed on from one generation to the next. There are two modes in which such a transmission of epimutations can occur (Skinner 2008):

- Multigenerational inheritance, in which several generations are affected because they all are exposed to the same factor (e.g., alcohol) and thus are prone to the same modifications; and
- Transgenerational inheritance, which involves a reprogramming event in the germline in response to a specific factor (e.g., alcohol exposure), resulting in an altered epigenome that would be inherited by future generations even if they are not themselves exposed to the same factor.

It was previously believed that transgenerational epigenetic inheritance would be unlikely because, as mentioned previously, epigenetic reprogramming occurs in the germline. However, increasing evidence indicates that transgenerational epigenetic inheritance does indeed happen (Anway et al. 2005; Crepin et al. 2012; Stouder and Paoloni-Giacobino 2010). Most of the work conducted thus far in this area has focused on the effects of agents that can interfere with the body’s normal hormone systems (e.g., vinclozolin, which affects sex hormone levels and has been shown to have transgenerational effects). The potential transgenerational effects of alcohol and their role in the etiology and perpetuation of FAS/FASD symptoms in affected individuals and their progeny, however, still need to be determined.

## Conclusions

**Figure 2:**
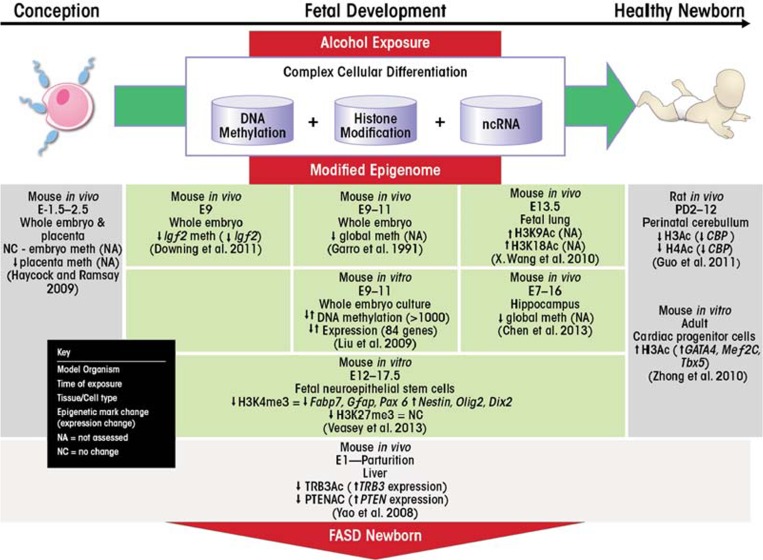
Epigenetic contributions to FASD. Following conception, a complex orchestration of epigenetic mechanisms ensures normal cellular differentiation and embryonic development (green horizontal arrow). These mechanisms include DNA methylation, histone modifications, and non-coding RNAs (ncRNAs) to modulate gene expression in a specified temporal and spatial manner. Alcohol exposure in utero (red downward arrow) has been shown to alter these epigenetic modulators, which may consequently dysregulate gene expression patterns as indicated by the study findings listed and affect normal embryonic development and phenotype outcome. By these mechanisms, alcohol-induced epigenetic aberrations may contribute to the etiology of fetal alcohol spectrum disorders (FASD).

Evidence is rapidly accumulating in support of an epigenetic etiology in the development of FASD (figure 2). All three types of epigenetic modulators—DNA methylation, histone modifications and regulation by ncRNAs—are perturbed by ethanol exposure. These ethanol-related changes can affect gene expression of critical developmental genes and pathways, impacting cell proliferation and differentiation.

The phenotypic consequences of in utero ethanol exposure are significantly correlated with the molecular consequences of ethanol’s effects on epigenetic regulatory mechanisms. A complex picture of locus-specific and cell-type–restricted effects is emerging. In particular, many studies have focused on ethanol’s effects on mechanisms that regulate neurogenesis, leading to the most devastating consequences of alcohol exposure during development. The range of effects appears to be significantly influenced by the timing and level of exposure, leading to a wide range of outcomes and combinations of phenotypic indicators.

In mouse models, ethanol exposure can be carefully controlled and other environmental parameters, such as diet and stress, can be kept constant. This allows for careful investigation of the effects of alcohol exposure on epigenetic regulatory mechanism and their association with FAS-like symptoms. Drinking patterns in pregnant women, in contrast, are seldom accurately documented and often occur throughout gestation, which, not surprisingly, leads to a vast array of phenotypes now recognized under the banner of FASD. Thus, discerning the role of epigenetic mechanisms in these processes will be much more challenging.

## Footnotes

1 Genomic imprinting is a genetic phenomenon in which only a gene copy inherited from one parent is expressed in the offspring. Each person inherits two copies of each gene, one inherited from the mother and one inherited from the father. In general, both of these gene copies can be active in the offspring. For some genes, however, one gene copy is “shut off” by genomic imprinting, so that only the non-imprinted copy remains active. For certain genes, the nonimprinted, active copy is always the one inherited from the mother, whereas for other genes the nonimprinted, active copy is always the one inherited from the father.
